# Endothelin A Receptors Expressed in Glomeruli of Renal Transplant Patients May Be Associated with Antibody-Mediated Rejection

**DOI:** 10.3390/jcm10030422

**Published:** 2021-01-22

**Authors:** Katarzyna Nowańska, Mirosław Banasik, Piotr Donizy, Katarzyna Kościelska-Kasprzak, Sławomir Zmonarski, Krzysztof Letachowicz, Dorota Kamińska, Oktawia Mazanowska, Hanna Augustyniak-Bartosik, Andrzej Tukiendorf, Anna Chudiak, Tomasz Dawiskiba, Agnieszka Hałoń, Magdalena Krajewska

**Affiliations:** 1 Department of Nephrology and Transplantation Medicine, Wroclaw Medical University, 50-367 Wrocław, Poland; nowanska@gmail.com (K.N.); slawomir.zmonarski@umed.wroc.pl (S.Z.); krzysztof.letachowicz@umed.wroc.pl (K.L.); dorota.kaminska@umed.wroc.pl (D.K.); oktawia.mazanowska@umed.wroc.pl (O.M.); hanna.augustyniak-bartosik@umed.wroc.pl (H.A.-B.); magdalena.krajewska@umed.wroc.pl (M.K.); 2 Department of Pathomorphology and Oncological Cytology, Wroclaw Medical University, 50-367 Wrocław, Poland; piotr.donizy@umed.wroc.pl (P.D.); agnieszka.halon@umed.wroc.pl (A.H.); 3 Research laboratory, Wroclaw Medical University, 50-367 Wrocław, Poland; katarzyna.koscielska-kasprzak@umed.wroc.pl; 4 Department of Public Health, Wroclaw Medical University, 50-367 Wrocław, Poland; andrzej.tukiendorf@umed.wroc.pl; 5 Division of Nursing in Internal Medicine Procedures, Department of Clinical Nursing, Faculty of Health Sciences, Wroclaw Medical University, 50-367 Wrocław, Poland; anna.chudiak@umed.wroc.pl; 6 Department of General, Vascular and Transplant Surgery, Wroclaw Medical University, 50-367 Wrocław, Poland; tomasz.dawiskiba@umed.wroc.pl

**Keywords:** endothelin A receptors, non-HLA antibodies, antibody-mediated rejection, allograft injury

## Abstract

Background: Non-human leukocyte antigen (HLA) anti-endothelin A receptor antibodies are presented as being potentially important, but the expression of the endothelin A receptor in glomeruli (ETA receptor (g+)) has not yet been described. We decided to evaluate the presence and relevance of the ETA receptor in for-cause renal transplant biopsies. The aim of our study was to evaluate the immunoreactivity of the ETA receptor and its significance in patients who underwent a renal transplant biopsy due to the deterioration of transplant function, with detailed characterization of staining in glomeruli. Methods: The immunohistochemical expression of ETA receptor (ETAR) was analyzed in renal transplant biopsies. Microscopic evaluation was performed on paraffin sections in glomeruli. The analysis was performed using a two-step scale (0: lack of ETAR expression; 1: the presence of ETAR expression—mild to moderate immunoreactivity). Results: We analyzed 149 patients who underwent renal allograft biopsy after renal transplantation. Positive staining of ETA receptors in glomeruli (ETA receptor (g+)) was noticed in 13/149 (8.7%) patients. Five of these 13 (38.5%) patients with ETA receptor (g+) developed antibody-mediated rejection (AMR), while 13 of the remaining 136 (9.5%) ETA receptor (g-) patients developed AMR (*p* = 0.0022). Graft loss was noticed in all but one ETA receptor (g+) patient with AMR (4/5; 80%), but only in 2/13 (15%) ETA receptor (g-) patients with AMR (*p* = 0.009) during the first year after biopsy. Conclusions: The expression of endothelin A receptors in glomeruli seems to be a potentially important feature in the diagnosis of damage during antibody-mediated rejection. It may help to identify patients at a higher risk of allograft rejection and injury.

## 1. Introduction

Humoral response is the main cause of graft loss, according to numerous sources of evidence [1,2,3,4,5,6,7,8]. The significance of anti-human leukocyte antigen (HLA) antibodies in transplantation is well described and acknowledged, but the role of non-HLA responses remains unclear.

Endothelin A receptor (ETA receptor) has recently been taken into account as one of the non-HLA antigens which may play an important role in immunological response and graft loss [9,10,11,12]. Endothelins (ETs) are peptides with a main function of vasoconstriction. These peptides are produced mainly in the endothelium [13]. Endothelin peptides include ET-1, ET-2, and ET-3 [14]. ET1 has been described as a factor which can possibly cause tubulointerstitial injury and proteinuria when it is produced in excess by the kidneys [15]. The proportion of endothelin A receptor (ETAR) to endothelin B receptor (ETBR) is about 9:1 in the renal artery and about 9. 2:8 in the renal vein. This suggests an essential role for ETAR in the regulation of renal vascular reactivity [16,17]. Wendel et al. also showed that receptors similar to human kidney ETARs are the predominant receptors that appear in rat renal vascular smooth muscle [18].

The presence of anti-ETAR antibodies is considered to be potentially unfavorable, but their role has not yet been established. Our previous research showed that the presence of anti-ETAR antibodies is connected with a worse transplant function when compared with a recipient without these antibodies [19].

The role of ETA receptors in renal transplant patients who had a for-cause biopsy was first described in our previous paper, proving that the expression of ETA receptors, evaluated in small and intermediate arteries of renal transplant tissue, was connected with acute tubular necrosis (ATN) or antibody-mediated rejection (AMR) [20].

In healthy conditions, non-HLA antigenic determinants are protected from circulating antibodies, but they are present and induce humoral response during transplant injury [21]. Humoral response may be present in patients with no anti-HLA antibodies [22]. More and more evidence indicates that ETA receptors are antigens that potentially induce immunization in patients with different transplanted organs [23,24,25,26].

Glomeruli are strongly involved in humoral response; hence, we decided to analyze the immunoreactivity of ETA receptors in this compartment of renal transplant biopsies which were performed in patients due to deterioration of graft function [27,28]. We believe that glomeruli play a crucial role in antibody-mediated rejection, and the staining of ETAR in these structures may be associated with injury. According to the newest Banff classification, chronic active AMR or chronic (inactive) AMR may be diagnosed when transplant glomerulopathy (cg > 0) is diagnosed. This motivated us to analyze the staining of ETAR in glomeruli and present our findings.

## 2. Methods

### 2.1. Patients and Sample Collection

Consenting patients undergoing a renal transplant biopsy with clinical indications as the standard of care between August 2011 and May 2016 were included. The indications for biopsy were described as deterioration in renal function (increase in creatinine of ≥0.3 mg/dl or proteinuria of ≥0.5 g/24 h) as a standard of care. Written informed consent was obtained from all patients. The patients were recruited from one center. The study was approved by the Wroclaw Medical University Research Ethics Board (KB-300/2018). All methods were performed in accordance with relevant guidelines and regulations.

### 2.2. Histopathology

Paraffin sections were prepared and assessed according to the Banff criteria. The pathologists (PD and AH) were unaware of the donor-specific antibody status. The presence of C4d depositions was assessed via the immunohistochemical method performed on paraffin sections using polyclonal antibody.

**Figure 1:**
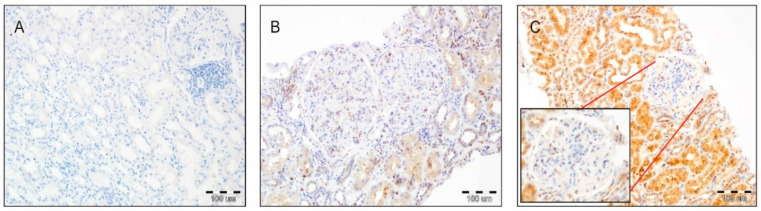
(**A**) Lack of endothelin A receptor (ETAR) expression in the tubular epithelium, glomeruli, and interstitium (200×, hematoxylin). (**B**) Mild to moderate expression of ETAR in glomeruli with mild to moderate reactivity in the tubular epithelium. Note the focal enhanced immunoreactivity of ETAR in the kidney interstitium (200×, hematoxylin). (**C**) Mild to moderate immunoreactivity in a glomerulus with high ETAR expression in the tubular epithelium (200×, hematoxylin).

The immunohistochemical expression of ETA receptors was analyzed in renal transplant biopsies. Microscopic evaluation of ETA receptor expression (rabbit polyclonal antibody, catalog number: G094 (P25101); dilution: 1:100; Assay Biotechnology Company, Fremont, USA) was performed on 4-µm-thick paraffin sections. These were mounted on silanized slides (DAKO, Glostrup, Denmark). ETA receptor expression was analyzed in the glomeruli of renal transplant biopsies. The analysis was performed using a two-step scale (0: lack of ETAR expression; 1: the presence of ETAR expression—mild to moderate immunoreactivity) (Figure 1).

Comparative co-analysis of the tubular epithelium and interstitium was performed on a three-step scale (0: lack of ETAR expression; 1: the presence of ETAR expression—mild to moderate immunoreactivity; 2: high ETAR expression).

### 2.3. Characteristics of Patients

Characteristics of the patients are shown in Table 1.

The initial immunosuppression involved tacrolimus or cyclosporine, mycophenolate mofetil, and steroids. In patients with higher panel reactive antibodies (PRA) (10–50) or a second transplantation, basiliximab was administered. Thymoglobulin was used if the PRA was above 50. Acute cellular rejection was treated using steroids, while antibody-mediated rejection was treated with plasmapheresis, intravenous immunoglobulins (IVIGs), and, occasionally, rituximab or bortezomib. The presence of donor-specific antibodies (DSA) was tested using solid-phase immunoassay technology (Luminex, Wroclaw, Poland).

### 2.4. Data Analysis

Statistica version 12 (Statsoft, Newport Beach, CA, USA) was used for statistical analysis. A *p* value below 0.05 was considered to indicate significance. The comparisons between baseline predictors and clinical outcomes were performed using Student’s *t*-test for parametric continuous variables and the Wilcoxon signed-rank test for nonparametric data. The Chi-squared and Fisher’s exact tests were applied to assess categorical variables. Univariate and multivariate logistic regression analyses were performed to evaluate the association of rejection risk factors with ETA receptor (g+) expression (Table 2).

We checked the influence of the number of grafts, the recipient’s age and sex, max PRA, the number of HLA mismatches, and anti-HLA antibodies on the presence of ETA receptor (g+). The association of ETA receptor (g+) with the presence of AMR was checked by applying univariate logistic regression.

## 3. Results

### 3.1. ETA Receptors in Glomeruli and Antibody-Mediated Rejection

We analyzed 149 consecutive patients who underwent renal allograft biopsy between 6 days and 23.5 years (median 643 days) after transplantation. Positive staining of ETA receptors in glomeruli was noticed in 13/149 (8.7%) patients. Five of these 13 (38.5%) patients with ETA receptors (g+) developed AMR, compared to 13 of the remaining 136 (9.5%) ETA receptor (g-) patients (*p* = 0.0023).

The association of ETA receptor (g+) with the presence of AMR was additionally verified by univariate logistic regression. We noticed a significant influence (*p* = 0.0055) (Table 3, Table 4 and Table 5).

### 3.2. Graft Loss with AMR in ETA Receptor (g+) and ETA Receptor (g-) Patients

Patients with ETA receptor (g+) who developed AMR lost transplants significantly more often (4/5, 80%) in comparison to patients with AMR but without ETA receptor expression in glomeruli (ETA receptor (g-); 2/13, 15%) (*p* = 0.009) during the first year after biopsy.

### 3.3. Expression of ETA Receptors in Glomeruli in Early Biopsies

Three out of 13 patients with positive glomeruli ETA receptor expression had early biopsies (during the first month), and the remaining 10 patients had late biopsies (beyond the first year after transplantation) (Table 3).

Two patients with early biopsy developed acute tubular necrosis. Both also had intense expression of ETA receptor in the tubular epithelium, described as high and assessed as 2 on the three-step scale. Both patients with acute tubular necrosis lost their transplants during the first year after biopsy.

### 3.4. Expression of ETA Receptors in Glomeruli in Late Biopsies

Ten patients with ETA receptor expression in glomeruli had late biopsies (14 months–12 years; median 4 years). Five of them (5/10, 50%) developed antibody-mediated rejection, and all but one lost their transplant during the first year after transplantation. All ETA receptor (g+) patients also had additional expression of ETA receptors in the tubular epithelium and interstitium. The expression was high (assessed as 2 in the three-step scale) in all but one patient in the tubular epithelium and in all but two patients in the interstitium (Table 3).

In comparison, ETA receptor (g-) patients were also characterized by low ETA receptor expression in the tubular epithelium and interstitium in late biopsies. There was no expression in the tubular epithelium in 23/69 patients and there was no expression in the interstitium in 46/69 patients.

### 3.5. AMR in Late Biopsies in Patients with and without ETA Expression in Glomeruli

A comparison of AMR development in late biopsies in patients positive and negative for ETA receptor in glomeruli showed a significant difference. AMR appeared in late biopsies in 5/10 (50%) ETA receptor (g+) patients, in comparison to 6/69 (8.6%) ETA receptor (g-) patients (*p* = 0.0004.)

### 3.6. ETA Receptor Expression in the Tubular Epithelium and Interstitium in ETA Receptor (g+) and (g-) Patients

The expression of ETA receptors in glomeruli was associated with the intensity of expression in the tubular epithelium and interstitium (Table 6).

In the ETA receptor (g+) group, all patients showed ETA receptor expression in the tubular epithelium and interstitium, while in ETA receptor (g-) patients, there was no expression in the tubular epithelium in 38/136 patients and no expression in the interstitium in 87/136 patients. In ETA receptor (g+) patients, in comparison to ETA receptor (g-) patients, a pathologist assessed the expression as high more often than low (2 vs. 1 according to the three-step scale employed) in the tubular epithelium (*p* = 0.001) and in the interstitium (*p* = 0.02).

### 3.7. Graft Loss in ETA Receptor (g+) Patients during the First 12 Months

Seven of the 13 (53%) ETA receptor (g+) patients lost their transplant during the first year after biopsy, while ETA receptor (g-) patients lost their transplant in 21/136 (15.5%) cases (*p* = 0.0007).

### 3.8. The Presence of Anti-ETAR Antibodies and DSA at the Time of Biopsy

The presence of anti-ETAR antibodies and DSA was checked at the time of biopsy. Positive results for anti-ETAR antibodies in ETARg+ patients were diagnosed in two cases. Four of the 13 ETARg+ patients had anti-HLA DSA.

## 4. Discussion

Our analysis indicated that the expression of ETA receptors in glomeruli may be related to damage during antibody-mediated rejection. Of the patients with ETA receptors in their glomeruli, 38.5% developed antibody-mediated rejection, compared to 9.5% of patients without ETA receptor expression in their glomeruli *(p* = 0.0022). Additionally, univariate logistic regression showed a significant impact of the presence of ETA receptors in glomeruli on the risk of AMR (*p* = 0.006).

We know that injury to microcirculation plays an important role in graft dysfunction. The presence of ETA receptor expression in glomeruli may suggest injury to this structure with the appearance of humoral injury and premature graft loss. Our analysis showed that significantly more patients with ETA receptor expression in their glomeruli lost their transplants early compared to patients without such ETA receptor expression: 53% vs. 15.5% (*p* = 0.0007). We also know from other sources that transplant glomerulopathy is the major cause of chronic graft dysfunction [29]. It was proved that 50% of patients with transplant glomerulopathy had donor-specific antibodies and poor graft survival [30]. Moreover, it is well known that transplant glomerulopathy is associated with humoral injury according to the Banff criteria [31]. Transplant glomerulopathy was first identified in the 1980s, with characteristic features defined as mesangial and endothelial cell changes in transplant kidney graft biopsies [32]. The key features observed in biopsy included duplication of the glomerular basement membrane, mesangial matrix expansion, and glomerulitis [33].

We do know that human leukocyte antigens, and, particularly, donor-specific antibodies against these antigens, play a crucial role in graft loss, but another target named non-HLA antigens (ETA receptor or AT1R receptor) may also be associated with increased incidence of antibody-mediated allograft rejection [34,35]. Pearl et al. underlined that autoantibodies similar to the closely related G-protein‒coupled receptor, anti-ETAR, are strongly associated with anti-AT1R antibodies [36]. In our previous study in 2014, we showed that anti-ETAR antibodies are associated with worse transplant function but also with histopathological features characteristic of antibody-mediated rejection. We indicated that anti-ETAR antibodies were connected with arteritis and vasculopathy [19]. In this analysis, we showed that the diagnosis of ETA receptor expression in glomeruli is significantly connected with antibody-mediated rejection.

The association of rejection risk factors with the presence of ETA receptor (g+) was checked via univariate and multivariate logistic regression analyses, and we did not notice any influence (Table 5). Antibody-mediated rejection is the main cause of graft loss [3]. Our analysis showed that patients who developed AMR significantly more often lost transplanted kidneys during the first year after biopsy when they developed ETA receptor expression in their glomeruli (ETA receptor (g+); 4/5, 80%) in comparison to patients with AMR but without ETA receptor expression (ETA receptor (g-); 2/13, 15%) (*p* = 0.009).

We are aware that there is still a trace of skepticism about the significance of autoantibodies in transplantation outcomes; nevertheless, a role for antigenic targets other than HLA in transplantation has been proposed [37,38,39]. The cause of uncertainty may be connected with slow progress in understanding the mechanism of injury, characterizing targets, developing useful detection tools, and, most of all, effective treatment [39]. However, there are more and more reports of antibody-mediated damage and graft loss in the absence of donor-specific HLA antibodies caused by antibodies against G-protein-coupled receptors (GPCRs), endothelin A receptor (ETAR), and angiotensin II type 1 receptor (AT1R) that cannot go unnoticed [40]. Lefaucheur et al. recently presented an excellent analysis of 1845 kidney transplant recipients, showing at the molecular level that the presence of anti-AT1R antibodies with transplant antibody-mediated injuries in biopsies is associated with increased expression of endothelial-associated transcripts (ENDATs) [35].

There are factors, such as inflammation, infection, or ischemia–reperfusion injury, which may affect receptor expression [39]. An inflammatory environment together with an enlarged amount of the proinflammatory cytokine immunoglobulin 6 (IL-6) is connected with increased AT1R expression on endothelial cells and may stimulate endothelial dysfunction [41]. Recurrent acute rejection may upregulate AT1R mRNA and, additionally, protein expression, which was described in biopsies of heart transplant recipients [42]. Increased ETA receptor expression may cause a breakdown of endothelial cell membrane integrity [43]. Increased expression of ETA receptor due to genetic differences or from stimuli may generate more targets to which antibodies can bind [39]. These factors may stimulate the autoimmune pathology observed when antibody-mediated rejection is reported [39].

Nowadays, one of the main challenges is diagnostic ambiguity in patients with histologic features of AMR without DSA, occurring in nearly half of cases [28]. In such situations, we should consider non-HLA antibodies, for example, anti-ETAR or anti-AT1R antibodies. There is certainly the question of therapeutic approaches which, in patients with AMR, are focused on removing circulating donor-specific antibodies, blocking their activity, and decreasing their production [44]. The identification of ETA receptor expression as a potential target for their antibodies may stimulate graft rejection and loss. It may have potential implications for clinical management and specific therapeutic strategies, which may selectively block ETA receptors. Future studies should also evaluate the usefulness of present therapies, such as plasma exchange and intravenous immunoglobulins (IVIg), which are the standard-of-care treatment for anti-HLA AMR [44]. We should not rule out the potential beneficial effect of anti-ETAR antibody removal during such a procedure [45]. Potential positive effects may be obtained using receptor antagonist [46,47].

We were not able to find a correlation of positive ETARg+ staining and anti-ETAR or DSA. However, we may not exclude the possibility that antibodies may join their receptors during active rejection and are thus not diagnosed in the serum.

We are conscious that our analysis of ETA receptors in glomeruli is a first observation and further analysis should be performed to confirm our findings. However, we should highlight that the expression of ETA receptors in glomeruli may be valuable for diagnosis and risk stratification. Antibody-mediated rejection is considered the most important cause of graft loss. We do not have a suitable treatment; therefore, the early diagnosis and identification of potential key antigens may be important in understanding their role in transplant injury. The recognition of ETA antibody-mediated injury may also be valuable for future treatment strategies to extend graft survival. There is, without doubt, a need for further research to understand the role of endothelin receptors after renal transplantation.

## 5. Conclusions

Our analysis showed that the expression of ETA receptors in glomeruli seems to be a potentially interesting feature in the diagnosis of injury during antibody-mediated rejection. Examining ETA receptors in glomeruli in addition to the current approach for immunologic assessment of kidney transplant recipients may help to identify patients with increased immunological risk. Further examination of ETAR’s significance is expected. The recognition of an injury with ETA receptor in glomeruli during rejection might potentially lead to the development of new diagnostic strategies.

## Figures and Tables

**Table 1:** Characteristics of the patients according to the presence of endothelin A (ETA) receptor in glomeruli.

Patient Characteristics	ETA Receptors in Glomeruli (+) *N* = 13	ETA Receptors in Glomeruli (-) *N* = 136	*p*
**Recipient age, years**	37.3 ± 16	43.5 ± 14	NS
**Male sex,** ***n*** **(%)**	8 (61.5%)	91 (66.7%)	NS
**Number of HLA ABDR mismatches**	3.58 ± 0.9	3.59 ± 1.1	NS
**A**	1.42 ± 0.5	1.34 ± 0.5	NS
**B**	1.25 ± 0.4	1.36 ± 0.6	NS
**DR**	0.9 ± 0.5	0.89 ± 0.6	NS
**Percentage of presensitized patients**			
PRA <10%	76,9%	82.2%	NS
PRA 10–50%	23,1%	13.5%	NS
PRA >50%	0%	4.3%	NS
**Living donors, No. (%)**	0/13 (0%)	8/136 (5.9%)	NS
**Deceased donors, No. (%)**	13/13 (100%)	128/136 (95.1%)	NS
**Retransplantation, No. (%)**	1/13 (7.7%)	5/136 (3.7%)	NS
**Cold ischemia time, hours**	24.3 ± 6.2	22 ± 8.9	NS
**Donor, male sex, %**	55%	58%	NS
**Donor age, years**	42.1 ± 13.4	48.5 ± 14.6	NS
**Cause of chronic renal failure:**			
Chronic glomerulonephritis	8	63	NS
Diabetic nephropathy	1	15	NS
Hypertonic nephropathy	0	19	NS
Polycystic kidney disease	1	15	NS
Pyelonephritis	1	8	NS
Others	2	16	NS
**Initial immunosuppression**			
Tacrolimus	8 (61.5%)	94 (69.1%)	NS
Cyclosporin	5 (38.5%	42 (30.9%)	NS
MMF/MPA	13 (100%)	133 (97.8%)	NS
azatioprine	0	3 (2.2%)	NS
anti-CD25 therapy	1 (7.6%)	5 (3.7%)	NS
**Time from the transplant to a biopsy, days**	1448 ± 1333	1612 ± 1933	NS
**Serum creatinine (mg/dl) at biopsy time**	2.53 ± 0.64	2.45 ± 1.02	NS

PRA—panel-reactive antibodies; HLA—human leukocyte antigen; MMF-mycophenolate mofetil/MPA-mycophenolic acid.

**Table 2:** Risk factors for ETA receptor (g+) patients (univariate and multivariate analysis).

Univariate Analysis				
	OR	−95% CI	+95% CI	*p*
No. of grafts	0.83	0.12	5.52	0.85
Recipient’s age	0.97	0.93	1.01	0.14
Male recipient	0.79	0.24	2.58	0.69
Max PRA	0.98	0.94	1.02	0.35
No. of MM HLA ABDR	1.20	0.85	1.72	0.29
anti-HLA Abs	1.40	0.44	4.49	0.56
		**Multivariate Analysis**		
	OR	−95% CI	+95% CI	p
No. of grafts	0.56	0.06	5.13	0.64
Recipient’s age	0.96	0.92	1.00	0.06
Male recipient	0.73	0.21	2.62	0.63
Max PRA	0.99	0.95	1.03	0.53
No. of MM HLA ABDR	1.09	0.62	1.93	0.74
anti-HLA Abs	1.46	0.41	5.23	0.56

OR, odds ratio; PRA, panel-reactive antibodies; MM, mismatch; No., number; HLA ABDR, human leukocyte antigen A, B, DR; Abs, antibodies.

**Table 3:** The association of positive expression of endothelin A receptor in glomeruli (ETA receptor (g+)) with the presence of antibody-mediated rejection (AMR) (univariate logistic regression).

Variable	OR	−95% CI	+95% CI	*p*
ETA receptor (g+) = 1	5.91	1.67	20.96	0.0055

**Table 4:** Analysis of patients with glomerular staining for ETA receptor expression using the Banff 2017 classification of renal allografts.

									Banff 2017 Classification					
Patient No.		ETAR Staining in Glomeruli	Time of Biopsy	Presence of DSA	g	i	t	v	ptc	C4d+	cg	ci	ct	cv
1	borderline changes	ETAR g+	early	no DSA	0	0	1	0	0	0	0	0	0	0
2	ATN, TCMR IA, IFTA 1	ETAR g+	early	no DSA	0	2	2	0	0	0	0	1	1	0
3	ATN	ETAR g+	early	no DSA	0	0	0	0	0	0	0	0	0	0
4	AMR, TCMR IB	ETAR g+	late	DSA	0	3	3	0	1	2	0	0	0	1
5	AMR, TCMR IB, IFTA 2	ETAR g+	late	DSA	0	2	3	0	0	1	3	2	2	3
6	AMR, IFTA 2	ETAR g+	late	DSA	0	0	0	0	0	0	3	2	2	3
7	AMR, IFTA 2	ETAR g+	late	DSA	0	1	2	2	3	3	3	2	2	3
8	AMR, TCMR IB	ETAR g+	late	DSA	1	2	3	0	0	0	1	0	0	0
9	TCMR IB, IFTA 1	ETAR g+	late	no DSA	0	2	3	0	0	0	0	1	1	0
10	TCMR IB, IFTA 2	ETAR g+	late	no DSA	0	2	3	0	0	1	0	2	2	0
11	TCMR IB, IFTA 2	ETAR g+	late	no DSA	0	2	3	0	0	0	0	2	2	0
12	TCMR IA, IFTA 2	ETAR g+	late	no DSA	0	2	2	0	0	1	0	2	2	0
13	IFTA 2	ETAR g+	late	no DSA	0	0	0	0	0	0	2	2	2	2

**Table 5:** Banff assessment of ETA receptor (ETAR)g+ and negative expression of endothelin A receptor in glomeruli (ETARg-) in patients.

	g	i	t	v	ptc	C4d+	cg	ci	ct	cv
ETARg+ AMR(+) [median (range)]	0 (0–1)	2 (0–3)	3 (0–3)	0 (0–2)	0 (0–3)	1 (0–3)	3 (0–3)	2 (0–2)	2 (0–2)	3 (0–3)
ETARg+ AMR(-) [median (range)]	0 (0)	2(0–2)	2 (0–3)	0 (0)	0 (0)	0 (0–1)	0 (0–2)	1,5 (0–2)	1,5 (0–2)	0 (0–2)
ETARg- AMR(+) [median (range)]	0 (0–2)	2 (0–2)	2 (0–2)	0 (0–2)	1 (0–3)	1 (0–3)	0 (0–2)	0 (0–2)	0 (0–2)	0 (0–2)
ETARg- AMR(-) [median (range)]	0 (0–2)	0 (0–3)	0 (0–2)	0 (0–2)	0 (0)	0 (0–2)	0 (0–2)	0 (0–3)	0 (0–3)	0 (0–3)

**Table 6:** ETA receptor expression in tubular epithelium and interstitium in ETA receptor g(+) and g(-) patients.

	Three-Step Scale	ETA Expression in Tubular Epithelium	ETA Expression in Interstitium
ETA receptor (g+) patients (*n* = 13)	at all	13	13
	2: high expression	11 *	9 **
	1: mild to moderate immunoreactivity	2 *	4 **
	0: lack of expression	0	0
ETA receptor (g-) patients (*n* = 136)	at all	98	49
	2: high expression	38 *	17 **
	1: mild to moderate immunoreactivity	60 *	32 **
	0: lack of expression	38	87

* high vs. low expression *p* = 0.001; ** high vs. low expression *p* = 0.02.

## Abbreviations:

AMR: antibody-mediated rejection; ATN: acute tubular necrosis; AT1 receptor: angiotensin II type 1 receptor; anti-AT1R antibodies: angiotensin type 1-receptor antibodies; anti-ETAR: antibodies, endothelin A receptor antibodies; ET: endothelin; ERK: extracellular signal-regulated kinases; ETA receptor: endothelin A receptor; ETA receptor (g+): positive expression of endothelin A receptor in glomeruli; ETA receptor (g-): negative expression of endothelin A receptor in glomeruli; GPCR: G-protein-coupled receptor; HLA: human leukocyte antibodies; OR: odds ratio; PRA: panel reactive antibodies.
